# Building a Low-Cost and Low-Fidelity Kidney Transplant Model: A Technical Report on the San Antonio Kidney Transplant Model

**DOI:** 10.7759/cureus.23883

**Published:** 2022-04-06

**Authors:** Ronit Patnaik, Mustafa T Khan, Seiji Yamaguchi, Danielle M Fritze

**Affiliations:** 1 General Surgery, University of Texas Health Science Center at San Antonio, San Antonio, USA; 2 Transplant Surgery, University of Texas Health Science Center at San Antonio, San Antonio, USA

**Keywords:** end-to-side anastomosis, vascular surgery, synthetic physical model, general surgery, transplant surgery, kidney transplant, simulation

## Abstract

One of the most challenging aspects of the kidney transplant operation is performing vascular anastomoses in the confines and depths of the iliac fossa. General surgery residents need to be adequately trained in this skill to maximize their intraoperative experience during their transplant surgery rotation. While several kidney transplant models have been developed, they are limited in their ability to simulate the challenges of performing anastomoses at varying depths and in confined spaces. Furthermore, they may be expensive or require specialized equipment, such as three-dimensional printers, to build. In this technical report, we describe how to build a low-fidelity, low-cost, and portable kidney transplant model capable of simulating vascular anastomoses at varying depths. Our model can be easily replicated for less than 30 USD using materials available in local stores. It uses inexpensive and reusable parts, allowing trainees a high volume of repetitions.

## Introduction

The technical skills of a transplant surgeon are paramount to achieving good patient outcomes in kidney transplantation. Birkmeyer et al. argue that technical proficiency might be one of the most important determinants of surgical outcomes [1]. For instance, the time and technical proficiency with which vascular anastomoses are performed during a kidney transplant influence the warm ischemia time of the donated organ. Prolonged warm ischemia time has been associated with worse long-term graft survival and patient outcomes [2-4]. One challenging aspect of performing vascular anastomosis during kidney transplantation is the depth and the confined space within the iliac fossa. Due to this time limitation and the degree of difficulty of the anastomosis, it is crucial that general surgery residents are adequately orientated and prepared before assisting in-vivo kidney transplantation during their transplant surgery rotation.

Simulated practice is widely considered to be a powerful tool to improve the technical skills of surgeons, particularly trainees. In 1993, Satava et al. described the first abdominal simulator to be used as a training tool [5]. Although numerous models allow trainees to practice vascular anastomoses, there remains a need for one that can adequately replicate the technical challenge of renal transplantation by incorporating variations in the depth and the confined space that is encountered in the iliac fossa. Such models should also be affordable, reusable, portable, and easily replicable.

In this technical paper, we describe the development of our low-cost, low-fidelity kidney transplant model capable of simulating the confined volume and various depths of the iliac fossa. This model is easily replicable and can be used by surgical training programs to orient and prepare their residents before performing in-vivo kidney transplants and to improve technical performance in kidney transplantation.

## Technical report

The San Antonio Kidney Transplant (SAKT) model can be built using household tools and replaceable items found in local stores. The list of items needed to build this model and their cost can be found in Table 1.

**Table 1:** Cost to build the renal transplant model. Items required to build include measuring tape, drill, and X-acto knife. *Arterial simulation required a polytetrafluoroethylene conduit. This was acquired from the local representative of the Gore company. They provided the expired grafts for free.

Item	Cost
Cardboard box	$1
Acrylic cylinder (6” diameter, 6” tall)	$15
Nuts ×6 (6/32”)	$1
Bolt ×6(6/32” × ½”)	$2
2” Alligator clips ×6	$8
Penrose drain	$2
Renal vein: 18” × ½” × ½”	
Iliac vein: 1” × 5/8” × 18”	
Arterial simulation*	
Total cost	$29

The following instruments were needed: vascular needle drivers, fine surgical forceps, two polypropylene sutures (we used 5-0 C1 double-arm), and two Shod clamps. If arterial anastomosis was being performed, participants used two 6-0 BV1 double-arm Prolene sutures.

**Figure 1:**
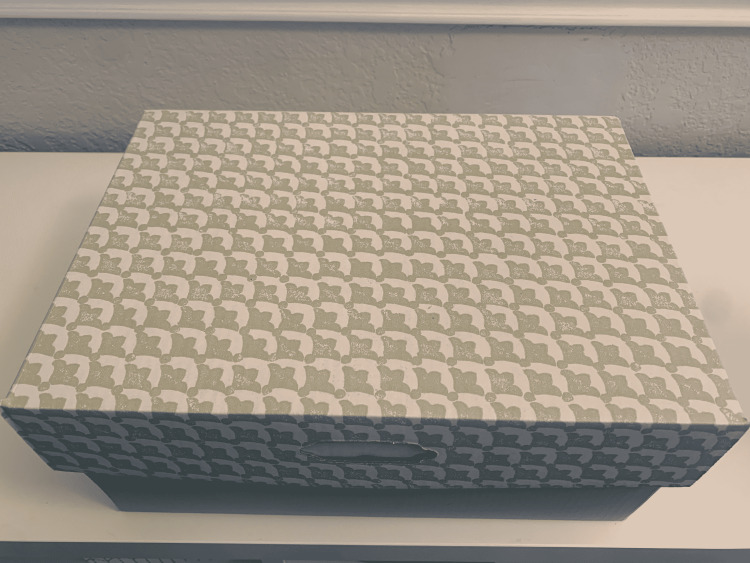
Cardboard box for the kidney model.

**Figure 2:**
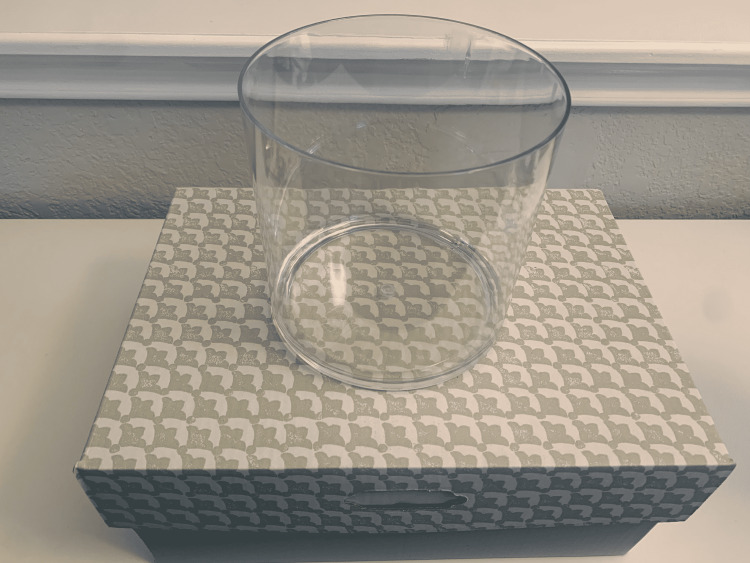
Use a cylinder to draw an outline on the top of the box.

**Figure 3:**
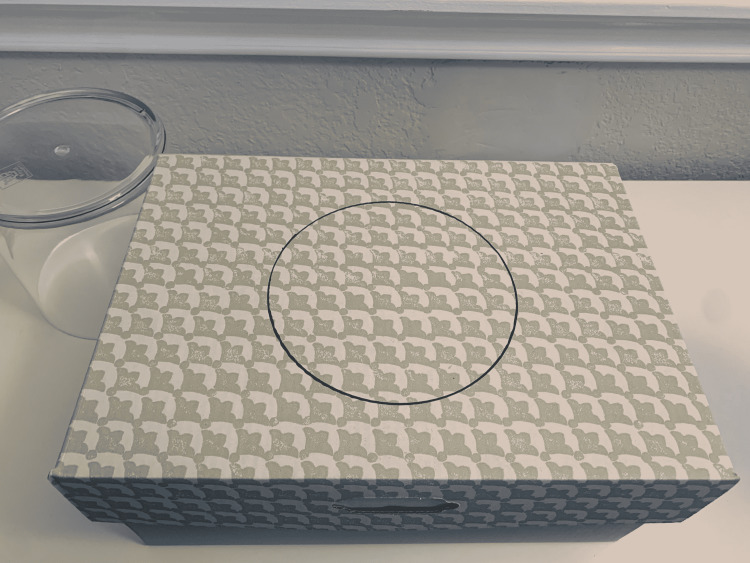
Circular outline on the top of the box.

**Figure 4:**
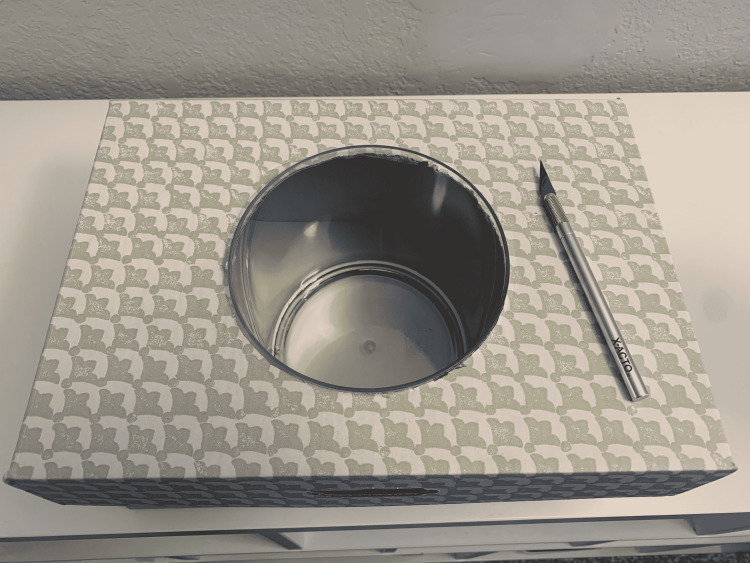
Place the cylinder within the box.

**Figure 5:**
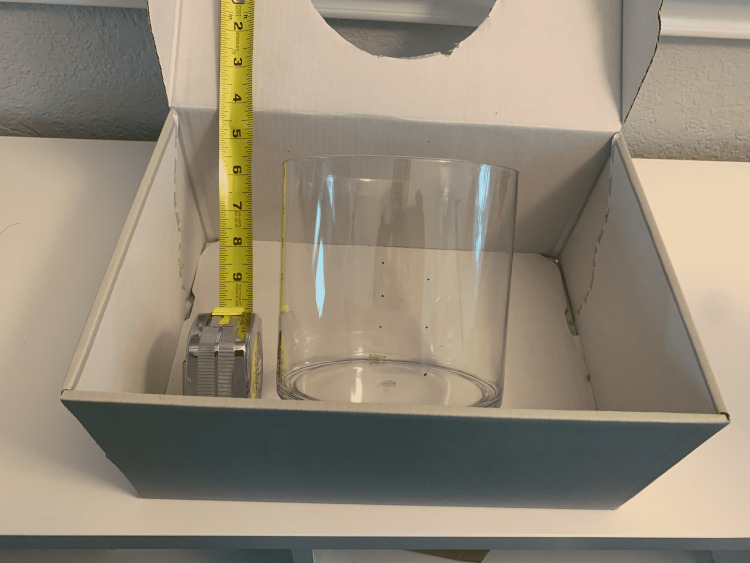
Measure and mark the acrylic cylinder.

**Figure 6:**
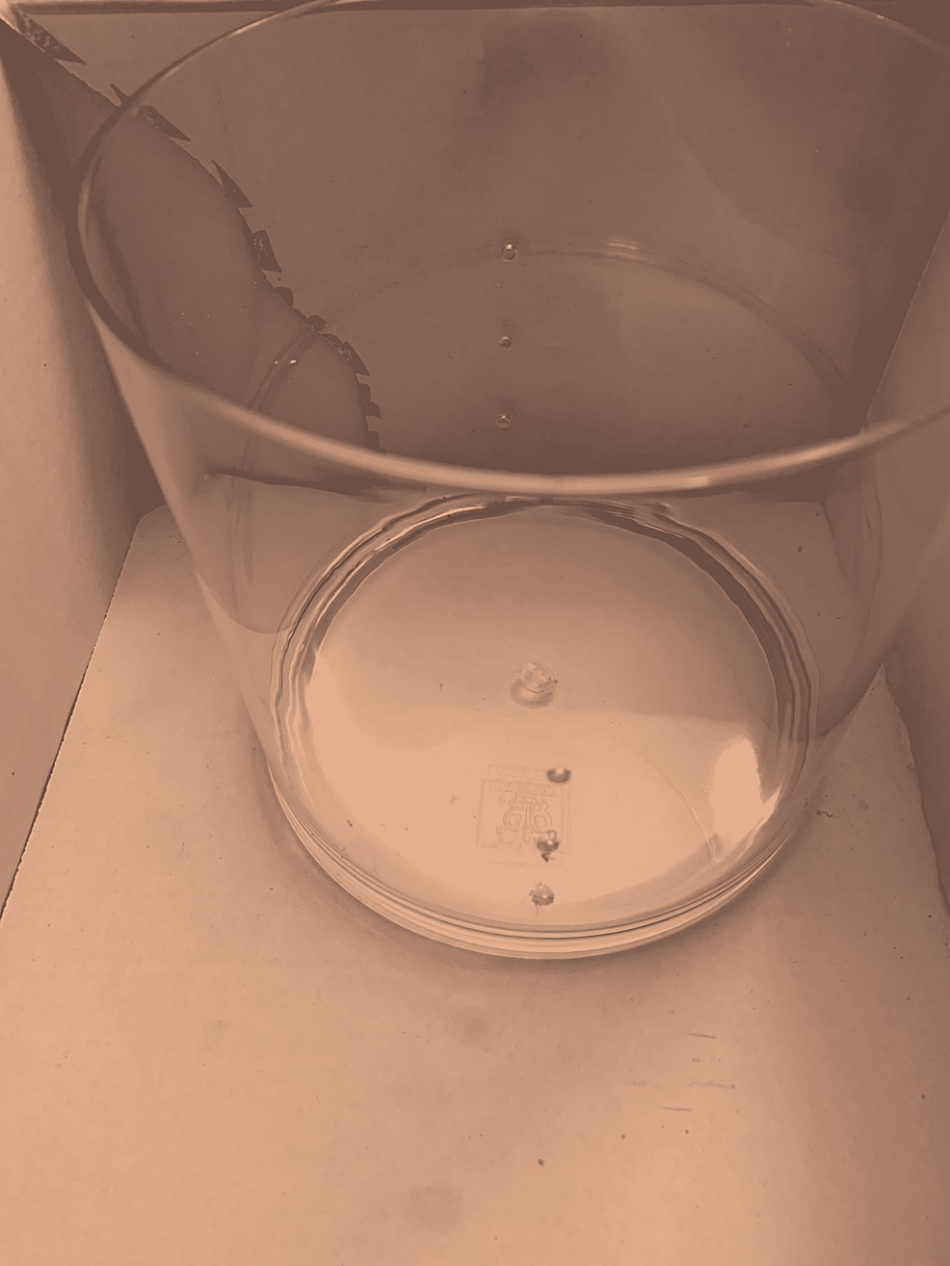
Drill initial holes.

**Figure 7:**
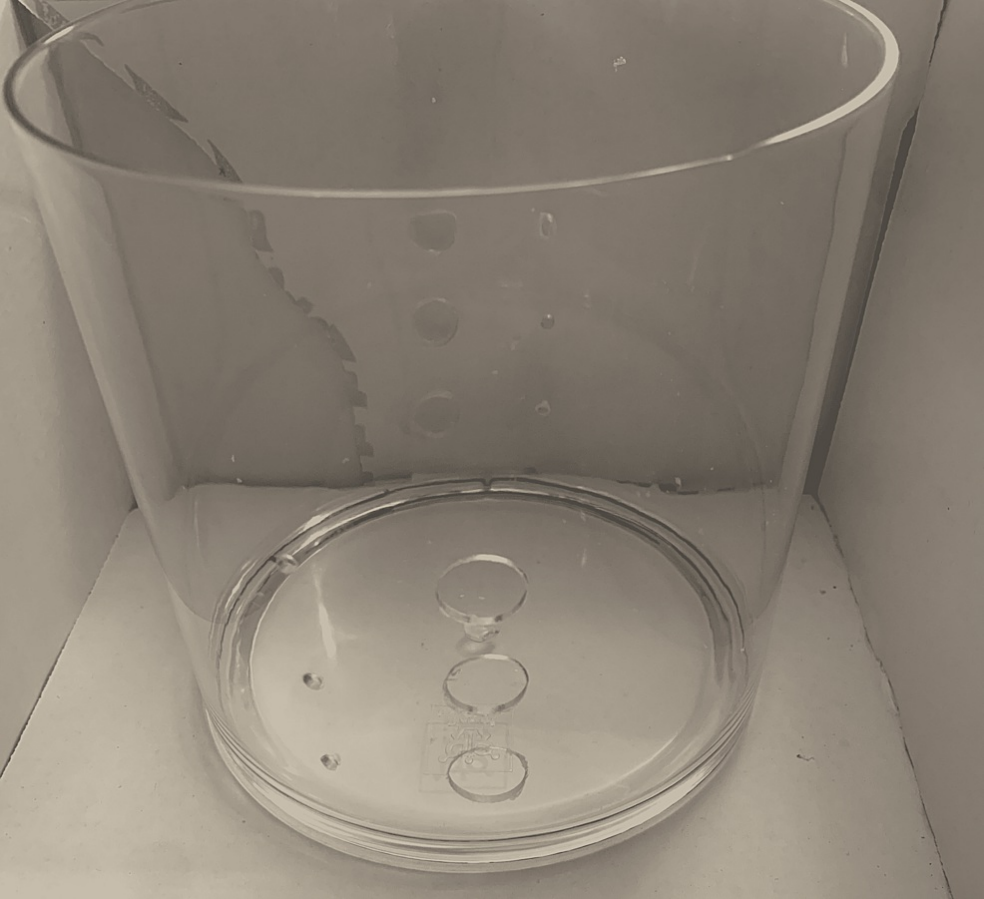
Up-size the initial holes using a larger drill bit.

**Figure 8:**
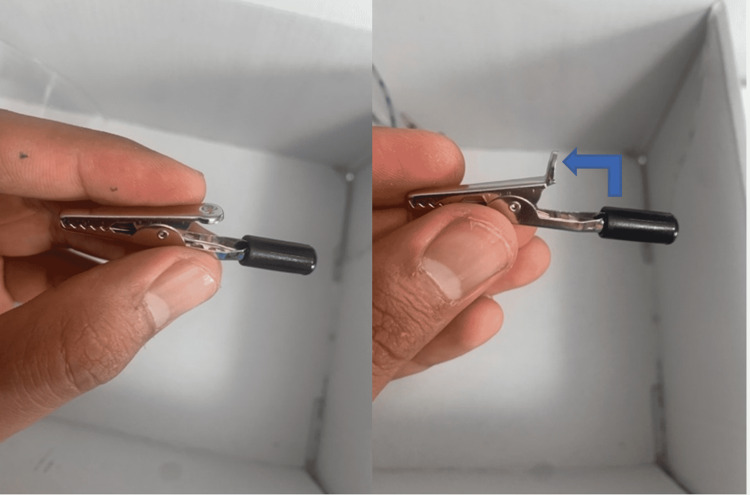
Preparing the alligator clip to use as clamps for the model.

**Figure 9:**
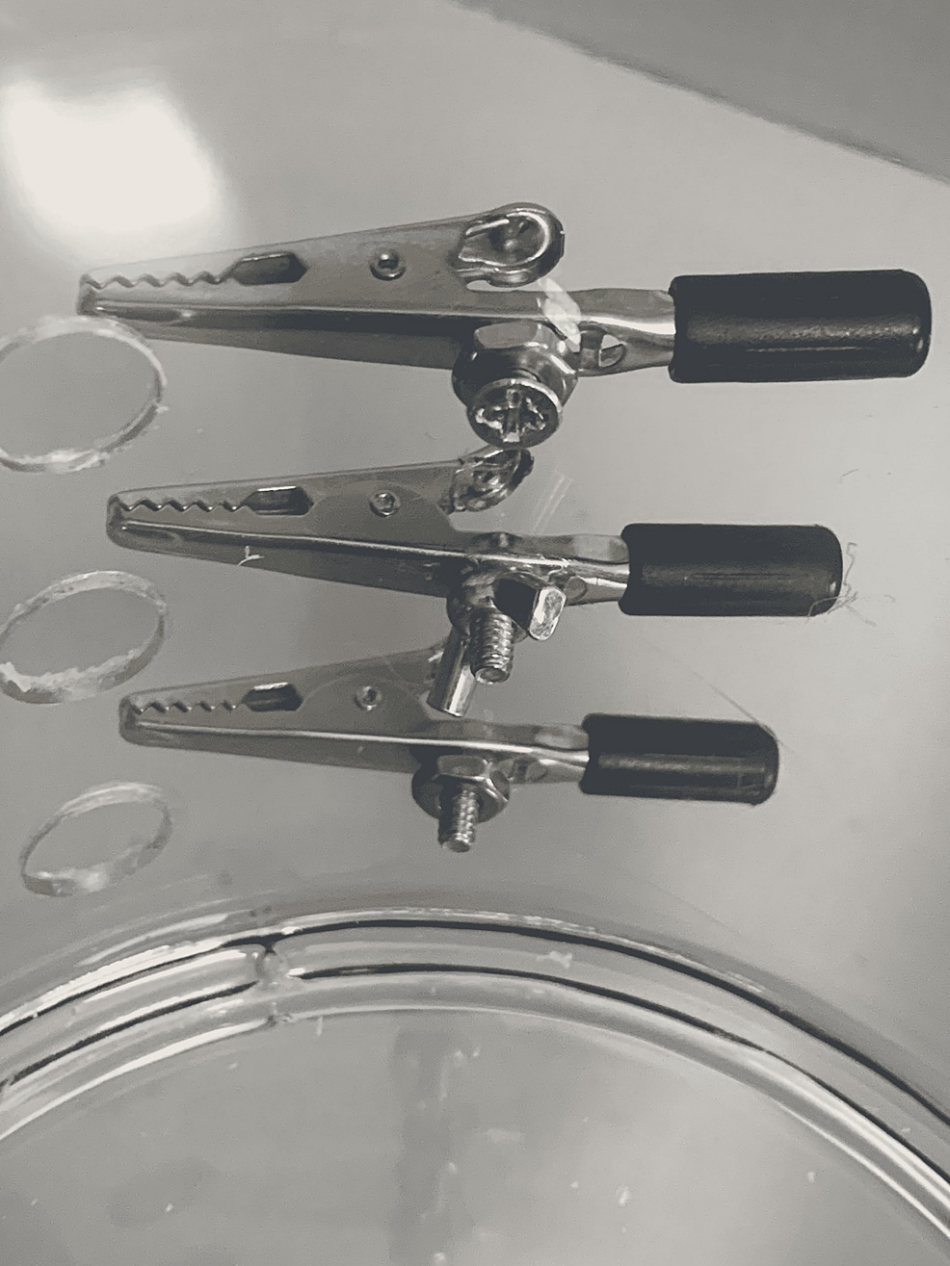
Attach alligator clips to the cylinder using nuts and bolts.

**Figure 10:**
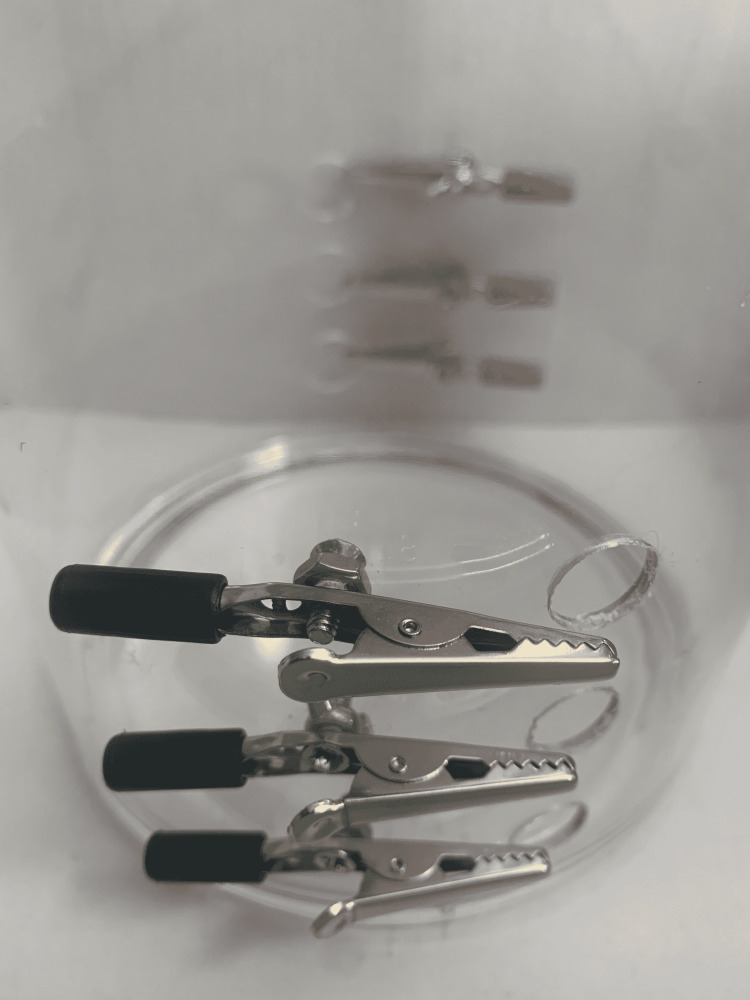
Ensure the alligator clips are attached on both sides.

**Figure 11:**
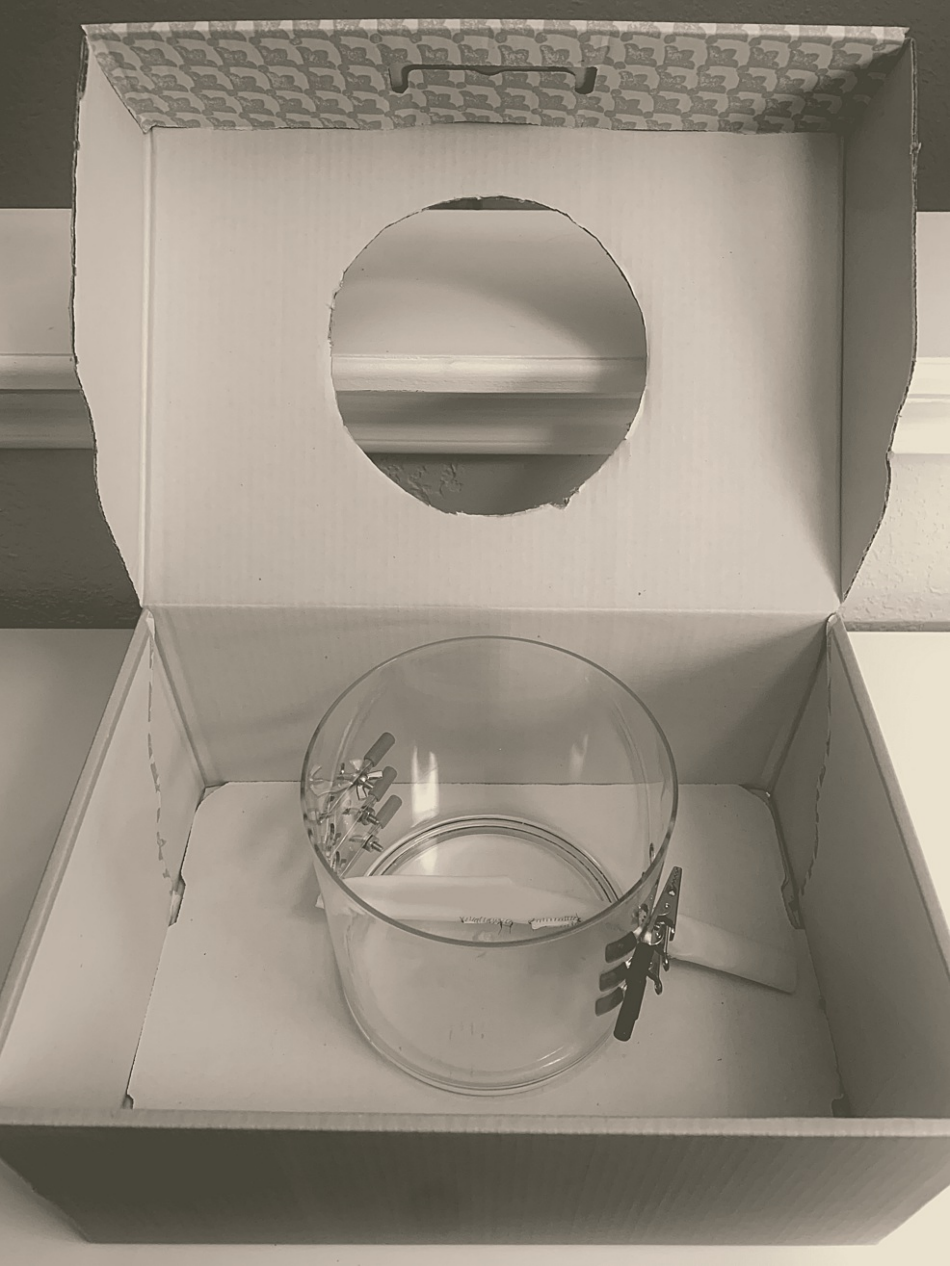
Suspend Penrose at the desired height.

**Figure 12:**
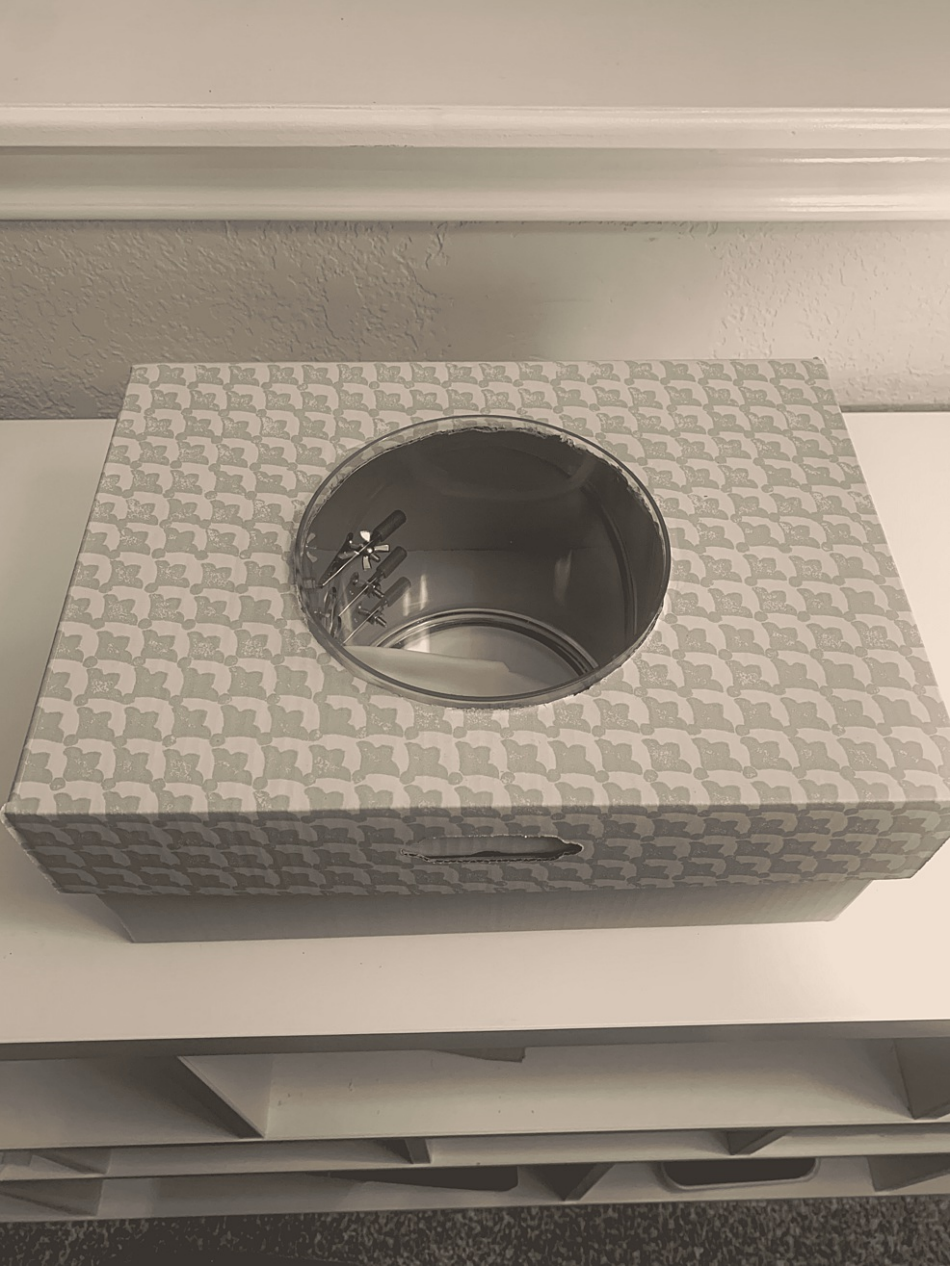
Assembled trainer.

**Figure 13:**
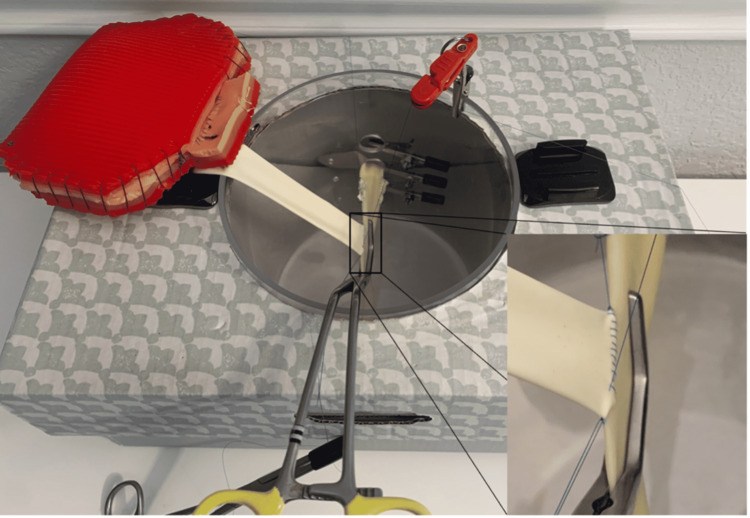
Assembled San Antonio Kidney Transplant model: venous anastomosis.

(1) Choose a cardboard box for your kidney model (Figure 1). The height of the box should be about the same or less than the acrylic cylinder.

(2) Use a 6-inch diameter acrylic cylinder to draw an outline on the top of the box (Figures 2, 3). Different cylinder sizes can be used for different skill levels and to add variety to practice.

(3) Using the circle, cut out the outline of the kidney model on the surface of the box. Then, the acrylic cylinder is placed within the box (Figure 4).

(4) Measure and mark the acrylic cylinder at 1-inch intervals starting at 2.5 inches from the top edge. Mark three spots on each side (180 degrees apart; Figure 5).

(5) Drill initial holes at the six marked spots (three on each side) using a 1/8” drill bit. Note that starting off with too large of a drill bit may damage the acrylic cylinder (Figure 6).

(6) Then, up-size the initial holes using 1/2” to 1” drill bits (Figure 7). The size of the holes will depend on the size of the Penrose used to practice these anastomoses.

(7) Use alligator clips to create the side clamps. Bend the tail of the clip 90 degrees away from the base. This will allow you to place the 6/32” screw through the hole in the base of the alligator clip (Figure 8).

(8) Screw the alligator clip onto the acrylic cylinder using 6/32” nuts and 6/32” bolts. Three variations are shown in Figure 9.

(9) Ensure the alligator clips are attached on both sides of the cylinder (Figure 10).

(10) Suspend Penrose at the desired height using the alligator clips and place the cylinder within the box (Figure 11).

(11) The assembled box trainer is shown in Figure 12.

(12) Set up instruments and the kidney for a simulated kidney transplant model (Figure 13). Figure 13 shows a renal vein to iliac vein anastomosis.

(13) In addition, this model can be used to simulate an end-to-side renal artery to iliac artery anastomosis. In this scenario, we used polytetrafluoroethylene (PTFE) as the vascular conduit. Because the PTFE was obtained for free from a local Gore company representative, we are unable to report a price (Figure 14).

**Figure 14:**
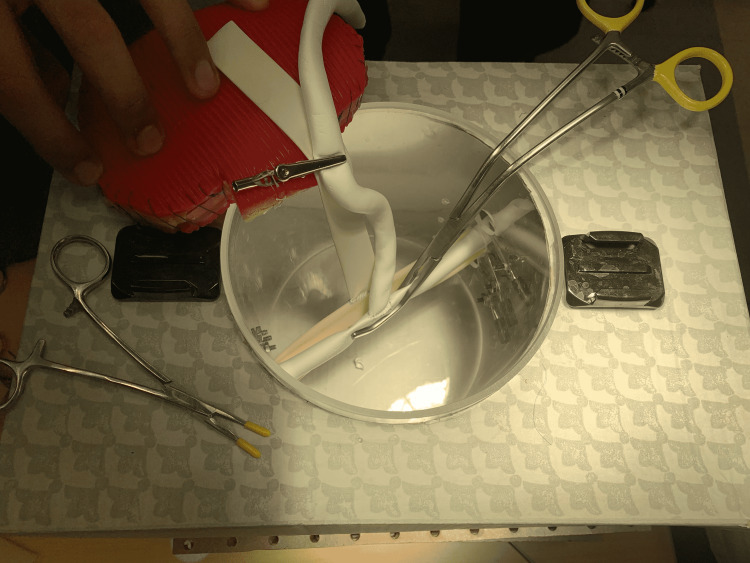
Setup for arterial and venous anastomosis.

**Figure 15:**
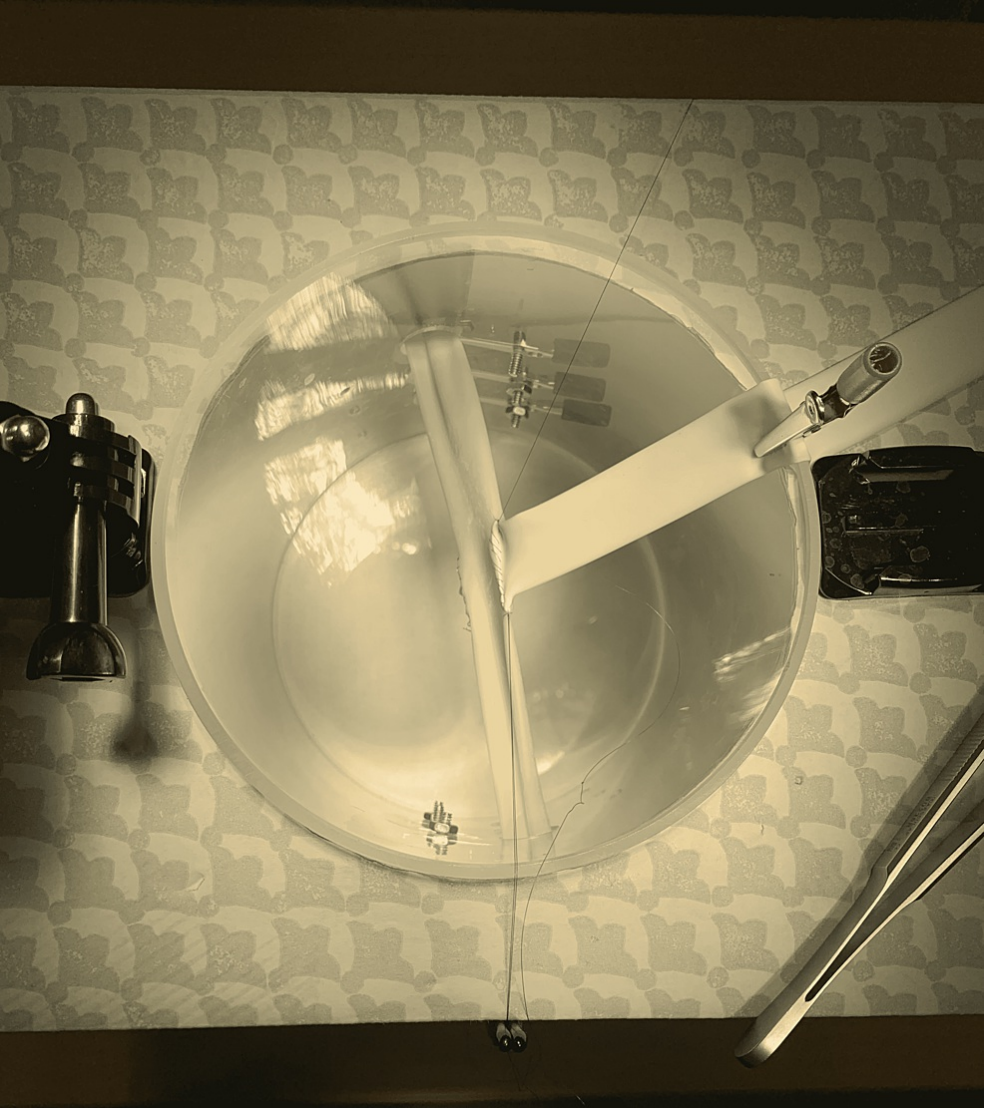
Setup for solo anastomosis practice.

(14) Finally, the model can be set up to practice a solo end-to-side renal vein to iliac vein anastomosis. This is not the ideal condition in which to practice; however, it allows the trainee flexibility to practice on their own (Figure 15).

## Discussion

The Accreditation Council for Graduate Medical Education (ACGME) requires a formal solid organ transplant experience for all general surgery trainees in the United States [6]. This rotation provides trainees with a unique blend of technical challenges and critical care experiences. Despite the efforts of training programs to incorporate transplant surgical experiences for their residents, the number of transplant procedures performed by graduating general surgery residents is limited, with a national average of 6.6 kidney transplants performed before graduation [7]. Moreover, because this procedure requires a high degree of technical skill at baseline, trainees may struggle to obtain adequate intraoperative experience if they are not well prepared with basic technical skills.

Particularly in low-volume, high-stakes procedures, simulation models can mitigate the paucity of intraoperative opportunities available for surgical trainees and augment their technical skills [8]. These models are used to facilitate the acquisition of procedure-specific skills outside the operating room by providing trainees with a low-stakes environment for practice [9].

Traditionally, the gold standard for simulation training in surgery, including transplant surgery, relied heavily on cadaveric and animal models [10-14]. While these models provided an excellent platform for realistic tissue and anatomy, their use has been limited by both cost (storage, maintenance, and disposal) and ethical dilemmas [15-17]. Furthermore, due to cost, these models are significantly limited in the number of practice sessions and repetitions they can provide to trainees.

Due to the limitations of both cadaveric and animal models, surgical educators in both general and transplant surgery have begun focusing their efforts on producing synthetic physical models that can represent a part or region of the body. These models can be used to practice and develop specific technical skills that represent certain critical portions of a surgical procedure. Low-fidelity task trainers are one such type of synthetic model that are often described in the literature as affordable and practical [18]. While these models do not attempt to closely replicate the look and feel of animate tissue, they provide a representation of specific portions of a procedure using affordable material that can be easily replaced for repeated practice.

In kidney transplantation specifically, previously proposed low-fidelity models consisted of various iterations utilizing small plastic boxes to mimic the iliac fossa and Penrose drains to mimic the vessels and ureter [19]. While these models are affordable and have proven to be useful in improving technical skills, they did not have the ability to represent the varying degrees of challenges associated with performing an anastomosis at increasing depth. On the other hand, three-dimensional printed kidney transplant models are resource-intensive to build, require a specialized printer and staff, and can be expensive for some institutions [20].

In this paper, we describe the materials and steps required to replicate our low-fidelity kidney transplant model. The primary benefit of this model is its ability to replicate the various depths of the human iliac fossa, thus allowing surgical trainees to become familiar with performing vascular anastomosis at various depths. Furthermore, this model is designed to replicate the technical difficulty of suturing in the confined space of the iliac fossa. Finally, while this model does not attempt to accurately replicate the look, feel, and behavior of animate tissue models, it provides a simple, affordable, and portable means to perform a high volume of repetitions that are often limited in more expensive models.

A limitation to this model is that to be used optimally, it requires an assistant to follow while practicing on it. However, it can be used by a single trainee; this adds a level of complexity that most novice trainees will take time to master. After using the model, the transplant surgery faculty at our institution reported that the model replicated a real renal vein anastomosis well and that it would add value to resident education, particularly before their first in-vivo kidney transplant. Refer to the Appendices for video demonstrations of how to use the SAKT model.

Future work should focus on obtaining validity evidence for this model and an objective scoring rubric. Furthermore, to be maximally effective, this model would need to be incorporated into a proficiency-based curriculum.

## Conclusions

In this technical paper, we describe a low-cost, low-fidelity, and replicable kidney transplant model designed to simulate the confined space and the varying depths of the iliac fossa. We hope that practicing on the SAKT model will allow trainees to increase their level of comfort in performing vascular anastomoses at varying depths and maximize their intraoperative learning and performance during in-vivo kidney transplantation.

## Appendices

Venous anastomosis with assistance

Arterial anastomosis with assistance
